# 小细胞肺癌骨转移的机制及其诊断标志物的研究进展

**DOI:** 10.3779/j.issn.1009-3419.2024.106.24

**Published:** 2024-09-20

**Authors:** Xingyu XIANG, Yandong NAN

**Affiliations:** 710038 西安，空军军医大学第二附属医院呼吸与危重症医学科; Department of Respiratory and Critical Care Medicine, Second Affiliated Hospital of Air Force Medical University, Xi’an 710038, China

**Keywords:** 肺肿瘤, 小细胞肺癌, 骨转移, 诊断, 标志物, Lung neoplasms, Small cell lung cancer, Bone metastasis, Diagnosis, Marker

## Abstract

小细胞肺癌（small cell lung cancer, SCLC）是一种恶性程度高、转变迅速、侵袭转移快的肺癌亚型，极易出现早期转移，预后较差。SCLC骨转移共经历癌细胞在原发部位增殖并突破局部组织、进入血液循环形成循环肿瘤细胞（circulating tumor cells, CTCs）并通过血液循环到达骨组织、到达骨组织后在骨微环境的支持下生根发芽形成新的肿瘤灶等三大阶段，而传统的影像学、病理学检查具有敏感性低、价格昂贵、实施难度大等缺点，基于血液标志物检测作为SCLC骨转移筛查与疗效评估的探索性研究近年多有报道。本文通过综述SCLC骨转移形成的分子生物学机制，发现SCLC骨转移中影像学及病理活检等常规诊断方法的不足，透明质酸、蛋白质生物标志物、非编码RNA、液体活检中的生物标志物等变化早于影像学变化，具有操作简便、可重复性好等优点，为SCLC骨转移早期诊断提供新思路、新方法，值得在临床推广应用。

## Abstract (en)

Small cell lung cancer (SCLC) is a type of lung cancer with high malignant degree, rapid transformation, rapid invasion and metastasis, which is prone to early metastasis and poor prognosis. Bone metastases of SCLC occur in three stages: cancer cells proliferate at the primary site, break through local tissues, enter the blood circulation to form circulating tumor cells (CTCs), reach bone tissue through blood circulation, and take root and germinate to form new tumor sites with the support of the bone microenvironment. However, traditional imaging and pathology examinations have disadvantages such as low sensitivity, high cost and difficulty in implementation. Exploratory studies based on blood marker detection as screening and efficacy evaluation of SCLC bone metastases have been reported in recent years. By reviewing the molecular biological mechanism of SCLC bone metastasis formation, this paper found that conventional diagnostic methods such as imaging and pathological biopsy were inadequate in SCLC bone metastasis. The changes in hyaluronic acid, protein biomarkers, non-coding RNA, and biomarkers in liquid biopsy were earlier than the changes in imaging, which had the advantages of simple operation and good repeatability. It provides a new idea and method for the early diagnosis of SCLC bone metastasis, which is worthy of clinical application.

肺癌是世界范围内发病率和死亡率最高的癌症类型，小细胞肺癌（small cell lung cancer, SCLC）是其中一种恶性程度高、转变迅速、侵袭转移快的亚型，占所有肺癌总数的15%-20%^[1]^。与非小细胞肺癌（non-small cell lung cancer, NSCLC）相比，其侵袭性更强，极易出现早期转移，预后较差。数十年来，SCLC早期仍缺乏有效的诊断方法且治疗手段相对局限，临床上SCLC的长期生存率仍然较低，尚缺乏标准的靶向药物，而骨转移作为SCLC患者常见的并发症之一，不仅患者生活质量较低，且治疗难度相对较大。因此，深入探索SCLC发生骨转移的分子生物学机制，从中探索新的诊断策略，早发现、早诊断骨转移显得尤为急迫和必要。目前，SCLC骨转移主要通过影像学和病理活检来确诊，均存在不足。理论上讲，在SCLC骨转移的过程中某些血液中的生物标志物变化早于影像学^[2]^，这些标志物的检测具有操作简便、可重复性好等优点，如果加以开发和利用，能够更便捷、更迅速地对SCLC骨转移进行早期诊断，从而对患者及早进行干预和管理。本文旨在综述SCLC骨转移的发病机制及诊断标志物的研究进展，以期为SCLC骨转移患者早期诊断提供线索。

## 1 SCLC骨转移的机制

SCLC骨转移的发病机制，目前通常借鉴“种子与土壤”学说^[3]^来解释。在这个理论中，种子一般指肿瘤细胞，土壤指肿瘤细胞迁移到的肿瘤微环境（tumor microenvironment, TME），是恶性肿瘤细胞赖以生存与发展的内环境。TME通常包含多种基质细胞，如免疫细胞、炎症细胞、成纤维细胞、平滑肌细胞和某些血管细胞等，是一个较为复杂的综合网络体系。而骨骼是中晚期恶性肿瘤常见的转移部位，在肺癌、乳腺癌及前列腺癌患者中尤其多见，可导致包括骨疼痛、病理性骨折、脊髓压迫、高钙血症等严重的骨相关事件（skeletal related event, SRE），不仅严重影响患者生活质量，甚至缩短患者生存期，晚期首诊肺癌患者骨转移发生率高达40%。如何早期、精确诊断肺癌骨转移，预防或避免相关SRE具有重要的临床意义。在SCLC发生发展过程中肿瘤细胞首先从原发肿瘤部位脱落，进入循环系统，随后迁移到骨骼。在骨骼中，肿瘤细胞与骨骼中的基质和细胞相互作用，通过分泌相关细胞因子和信号分子来影响骨的代谢过程，进而导致骨质的破坏和肿瘤细胞的增殖，最终形成转移性骨肿瘤。

### 1.1 原发肿瘤的局部侵袭

SCLC肿瘤细胞首先在肿瘤原发部位增殖，然后通过改变细胞黏附分子如E-钙黏素^[4]^来减弱肿瘤细胞间的黏附力，增强肿瘤细胞的转移能力。其次，SCLC转移的过程涉及到多种蛋白水解酶表达的变化，这些酶参与了细胞外基质的降解，是肿瘤细胞侵袭和转移的关键^[5]^。Rath等^[6]^研究了包括基质金属蛋白酶（matrix metalloproteinase, MMPs）、解聚蛋白样金属蛋白酶（a disintegrin and metallo-proteinases, ADAMs）、组织蛋白酶（Cathepins）、碱性蛋白酶（Kallikreins）等在SCLC细胞系中的表达情况，检测出这些酶的高表达能够降解细胞外基质成分，从而为肿瘤细胞转移提供肥沃的土壤。

### 1.2 肿瘤细胞进入血液循环

循环肿瘤细胞（circulating tumor cell, CTCs）是脱落于原发肿瘤并进入循环系统的细胞，它们在多种恶性肿瘤转移中起着至关重要的作用^[7]^。CTCs同样也在SCLC侵袭转移中扮演重要角色。Micalizzi等^[8]^在研究中指出，CTCs通过改变它们的细胞表型、与血管壁和其他细胞的相互作用来适应和操控血液循环，而CTCs的形状和可塑性等生物力学特征也是SCLC侵袭转移的重要因素。此外，CTCs可以通过表达血小板反应蛋白-1（thrombospondin-1, TSP-1）、尿激酶型纤溶酶原激活受体（urokinase-type plasminogen activator defibrase capacity receptor, uPAR）、血管内皮生长因子（vascular endothelial growth factor, VEGF）和血管生成素-2（angiopoietin-2, Ang-2）来促进新血管的形成^[9]^，以获取更多的氧气和养分，为肿瘤的扩散提供通道。

### 1.3 肿瘤细胞在骨骼中定居

CXC趋化因子受体4（C-X-C chemokine receptor 4, CXCR4）与多种癌症的生长和转移有关，包括SCLC^[10]^。而CXC趋化因子配体12[chemokine (C-X-C motif) ligand 12, CXCL12]作为趋化因子，在骨髓中高表达^[11]^。Taromi等^[12]^观察到，表达CXCR4的肿瘤细胞向CXCL12迁移，而CXCR4抑制剂能抑制SCLC肿瘤生长和转移形成，这表明SCLC细胞通过表达特定的细胞表面受体CXCR4与骨髓中的趋化CXCL12相结合，导致肿瘤细胞在骨骼中定居。此外，整合素作为一类重要的细胞表面受体，负责调节细胞与细胞外基质之间的黏附^[13,14]^。在SCLC肿瘤细胞中特定的整合素亚型如β1、β3的增加^[15,16]^，有助于肿瘤细胞附着在骨基质上，促进骨转移的形成。

### 1.4 骨微环境与肿瘤细胞的相互作用

在骨微环境中转移性肿瘤细胞和骨基质细胞之间的相互作用，为肿瘤细胞提供了肥沃的土壤。转化生长因子-β（transforming growth factor-β, TGF-β）可刺激破骨细胞的诱导和分化，同时也在转移性肿瘤细胞的增殖中起关键作用^[17]^。TGF-β被破骨细胞吸收后从骨基质中释放出来，增加了甲状旁腺激素相关肽（parathyroid hormone-related peptide, PTHrP）的表达，而PTHrP能刺激破骨细胞活化，导致骨破坏^[18]^。骨破坏后又释放出TGF-β^[19]^，进一步刺激肿瘤细胞的生长和分化，形成一种恶性循环。作为TGF-β的超家族成员，骨形态发生蛋白（bone morphogenetic protein, BMP）也对肿瘤细胞的骨转移发挥了重要作用。Huang等^[20]^通过研究发现骨形态发生蛋白-2（bone morphogenetic protein 2, BMP2）可以增强巨噬细胞向破骨细胞的分化，表明BMP2信号传导有助于溶骨转移；与此同时，他们还发现BMP2能诱导成骨细胞分化，成骨细胞机制也可能在肺癌的骨转移中发挥作用。此外，RANKL/RANK通路亦可控制破骨细胞募集、多核破骨细胞的融合、破骨细胞活化和破骨细胞存活^[21]^，RANKL的过表达与转移性溶骨性骨骼病变相关，从而促进SCLC转移性骨骼病变^[22]^。

### 1.5 免疫系统对肿瘤细胞的影响

当SCLC肿瘤细胞转移到骨骼时，机体免疫系统与肿瘤细胞之间会相互影响。众所周知，免疫系统的主要功能是识别并清除体内异常细胞，包括肿瘤细胞。然而，在SCLC骨转移的情况下，免疫系统可能会受到多种因素的影响，影响了其对抗肿瘤细胞的效能。首先，SCLC存在肿瘤组织中的浸润淋巴细胞少、肿瘤组织程序性死亡配体1（programmed cell death ligand 1, PD-L1）表达阳性率低、主要组织相容性复合体表达水平低，且具有抑制型免疫细胞如肿瘤相关巨噬细胞、髓系衍生抑制细胞等的特性^[23]^，以此来逃避免疫系统的攻击。其次，当SCLC癌细胞转移到骨骼时，它们会破坏骨基质并释放诸如吲哚胺2,3-双加氧酶-1（indoleamine-2,3-dioxygenase 1, IDO1）、白细胞介素10（interleukin-10, IL-10）等免疫抑制剂及调节性T细胞（regulatory T cells, Treg）等^[24]^化学物质，这些物质会干扰免疫细胞的正常功能。在治疗过程中，垂死的肿瘤细胞中释放损伤相关分子如腺嘌呤核苷三磷酸（adenosine triphosphate, ATP）和高迁移率族蛋白B1（high mobility group protein-1, HMGB1）亦可刺激IL-1α和其他免疫刺激细胞因子的产生，导致免疫抑制^[25]^，进而促进肿瘤进展和耐药的产生。由此可见，抗肿瘤的关键是要让免疫系统先识别出肿瘤细胞，才能调动其他的免疫系统细胞一起来抗肿瘤，而程序性死亡受体1（programmed cell death 1, PD-1）/PD-L1抑制剂、细胞毒性T淋巴细胞相关蛋白-4（cytotoxic T-lymphocyte antigen-4, CTLA-4）抗体和靶向整合素相关蛋白（integrin-associated protein, IAP/CD47）的单抗/融合蛋白药物都是很好的利器。这些药物进入体内后，可以阻止肿瘤细胞的免疫逃逸作用，帮助免疫系统准确识别“坏细胞”，从而重新激活肿瘤患者免疫系统的抗肿瘤反应，而当下热门的嵌合抗原受体T细胞免疫疗法正是通过增强T细胞靶向杀伤肿瘤细胞的能力来达到抗癌的目的。

综上，SCLC骨转移具有特异性及土壤依赖性、肿瘤细胞起源、基因状态改变、细胞间的信号交流及TME等多种因素共同促成了其强侵袭转移能力的生物学特征。曾有学者^[24]^总结了临床前模型在SCLC中的临床应用进展，并根据器官转移趋向性特点对转移相关的细胞表型及分子机制进行归纳后发现，上皮间质转化（epithelial-mesenchymal transition, EMT）及干细胞相关表型均与SCLC侵袭转移生物学行为相关。此外，SCLC 转移过程具有器官趋向性，多数患者会发生脑转移、肝脏转移及骨转移，目前对SCLC器官特异性转移机制的理解仍非常有限，有关血管生成因子、核因子IB（nuclear factor IB, NFIB）等因素具有器官特异性作用还是能普遍促进多脏器转移还需要进一步研究确定。如图1所示，SCLC细胞骨转移共经历三个阶段：（1）癌细胞在原发部位增殖并突破局部组织；（2）进入血液循环形成CTCs，通过血液循环到达骨组织；（3）到达骨组织后，在骨微环境的支持下生根发芽，形成新的肿瘤灶。每个步骤都涉及了众多信号通路和分子，且各信号通路和分子相互交织，在各个阶段中发挥作用，同时在免疫系统的影响下最终形成了骨转移。

**图1:**
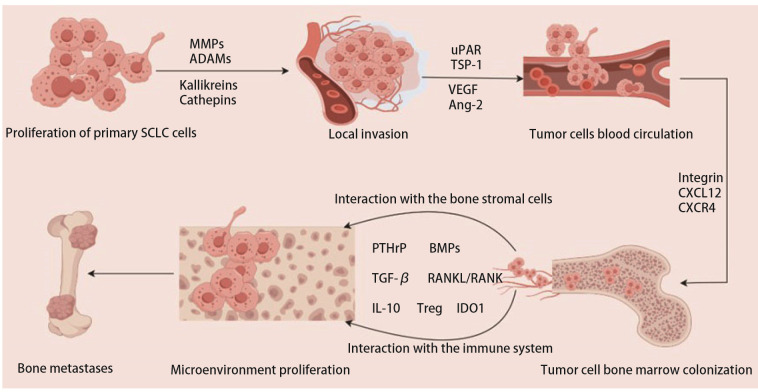
SCLC细胞的骨转移途径

## 2 SCLC骨转移诊断的生物标志物

肺癌较易出现远端转移，骨骼则是其常累及的器官之一。据统计，大约有2/3的SCLC患者在初次诊断时即伴有远处转移，而仅有1/3的患者病灶局限在胸部^[26]^。目前，肺癌血运转移具体机制尚未完全清楚，但血运转移的先决条件是有足够多的肿瘤细胞突破基底膜入血并随血液循环带至全身各处。肿瘤细胞入血必然使血清中的相关肿瘤标志物的浓度升高，而且这种升高同入血的肿瘤细胞数量存在着相对正相关。因此，探索新的骨转移生物标志物在SCLC骨转移初步诊断中愈发重要。

### 2.1 透明质酸（hyalurniaid, HA）

HA是一种酸性黏多糖，是细胞外基质的基本元素之一，参与各种肿瘤转移的发生。HA与CD44结合后通过激活各种信号转导通路（如Rho GTP酶、Ras-MAPK和PI3K/AKT通路）在调节细胞黏附、迁移、存活和侵袭方面发挥重要作用，干扰HA/CD44通路会限制肿瘤生长和转移^[27]^。Zhao等^[28]^通过多因素Logistic回归分析了SCLC骨转移患者血浆中的HA和CD44，发现HA是骨转移的独立预测因素，HA可以稳定可靠地预测SCLC中的骨转移。

### 2.2 蛋白质生物标志物

蛋白质生物标志物最常用于肿瘤骨转移的临床诊断和预后。血液中蛋白质的存在或异常表达通常与某些类型的肿瘤有关。这些蛋白质可以在肿瘤细胞、周围组织和血液中检测到。

#### 2.2.1 膜联蛋白-A1（annexin A1, ANXA1）

ANXA1是膜联蛋白超家族的成员，以钙依赖性方式与酸性磷脂结合。ANXA1由癌症相关成纤维细胞分泌，可以增加癌症干细胞的生成，目前已被证实参与肿瘤细胞增殖、血管生成、迁移、侵袭，而且是肿瘤来源性细胞外囊泡的关键组分^[29]^。Chen等^[19]^通过对SCLC细胞系的研究表明ANXA1过表达增强了SCLC细胞系的增殖、迁移和侵袭，通过检测SCLC骨转移患者血清中的ANXA1水平确定了ANXA1的表达与骨转移之间的关联。

#### 2.2.2 糖蛋白非转移性黑色素瘤蛋白B（glycoprotein non-metastatic melanoma protein B, GPNMB）

GPNMB也称为造血生长因子诱导型神经激肽1和树突状细胞相关硫酸肝素蛋白多糖依赖性整合素配体，是一种由572个氨基酸组成的I型跨膜蛋白^[30]^。与正常肺相比，GPNMB在SCLC肿瘤组织中普遍存在^[31]^。Liu等^[32]^曾收集88例SCLC患者的血浆，采用酶联免疫吸附测定GPNMB在其血浆中的表达水平，结果显示广泛期SCLC组血浆GPNMB平均浓度明显高于局限期SCLC组，其中骨转移组、高基线血浆GPNMB患者的总生存期（overall survial, OS）较短（P=0.0299），且抑制GPNMB的表达可有效降低SCLC的转移和增殖能力。因此，检测GPNMB水平或可用于指导SCLC患者的个性化治疗。

#### 2.2.3 基质金属蛋白酶-9（matrix metalloproteinase-9, MMP-9）

MMP-9能降解细胞外基质中的蛋白成分，破坏肿瘤细胞侵袭的组织学屏障，在肿瘤侵袭转移中起关键性作用。Li等^[33]^分析了MMP-9在SCLC患者肿瘤组织中的阳性表达率为70.8%，高表达与病理分期相关。MMP-9在肿瘤组织中的表达和活性显著升高，支持了这种金属蛋白酶在SCLC生长和转移中的重要作用。此外，Zhang等^[34]^在一项纳入了240例受试者的病例对照研究中发现SCLC组患者血清MMP-9表达水平显著高于健康对照组，且其表达水平与肺癌的临床分期、分化程度、淋巴结转移、吸烟史及组织病理类型密切相关，表明MMP-9在肺癌病情评估方面起着重要作用。Xu等^[35]^的研究结果也证明了这一观点，同时还发现可以通过沉默TRIM66逆转过表达的MMP-9对SCLC细胞迁移、侵袭和EMT的影响。

### 2.3 非编码RNA

随着生物信息学的发展，人们发现大量非编码RNA如微小RNA（microRNA, miRNA）、长链非编码RNA（long non-coding RNA, lncRNA）和环状RNA（circular RNA, circRNA）参与基因表达调控、细胞分化等，它们与肿瘤的发生发展也密切相关。Zhang等^[36]^利用下一代测序（next-generation sequencing, NGS）技术发现29个lncRNA、48个miRNA和510个信使RNA（messenger RNA, mRNA）差异性表达，通过生物信息学筛选出4个lncRNA和miRNA，并通过定量聚合酶链反应（polymerase chain reaction, PCR）进行验证，分析了这些非编码RNA与SCLC发生和转移的相关性。这些信息可能有助于我们完善SCLC的诊断和管理。一些其他研究^[37]^也证实了SBF2-AS1（一种lncRNA）在SCLC细胞系的表达升高，SBF2-AS1的高表达与SCLC患者的临床分期、肿瘤大小、远处转移有关，SBF2-AS1高表达是SCLC患者OS的独立不良预后因素。此外，circVAPA（一种circRNA）可以通过调节miR-377-3p和miR-494-3p/胰岛素样生长因子1受体（insulin-like growth factor 1 receptor, IGF-1R）轴来激活PI3K/AKT信号通路，在细胞系和人体中加速SCLC进展和转移^[38]^。

### 2.4 液体活检中的生物标志物

液体活检主要从血液、尿液等体液中筛选与骨转移相关的分子指标，包括循环肿瘤DNA（circulating tumor DNA, ctDNA）、CTCs和外泌体（exosome）。与传统的组织样本活检相比，液体活检标志物具有以下优势：（1）无创：无需组织切除和穿刺，仅需提取血液或尿液；（2）全身性：液体样本能反映全身情况，避免组织样本采集中的局部误差；（3）灵敏度高：液样中标志物浓度相对稳定，不受组织异质性影响，使检测结果更加准确可靠；（4）重复性好：液体样本采集相对简单、无创，可多次采集，监测肿瘤生长和转移；（5）前瞻性：在早期肿瘤的检测和监测中，液体活检可以提供一种更加灵活灵敏的检测方法，提高肿瘤的早期诊断和治疗率。

#### 2.4.1 CTCs

CTCs在大多数SCLC患者血液中均可检测到，通常数量相对较高^[39]^。Welsch等^[40]^通过使用微流控或密度梯度CTCs富集与免疫荧光染色或CTCs相关转录本的实时定量基因扩增荧光检测系统（real-time quantitative PCR detecting system, qPCR）相结合检测了EpCAM、CK19、突触素（synaptophysin, SYP）、嗜铬粒蛋白A（chromogranin A, CHGA）和Notch信号通路Delta样配体3（delta-like 3 protein, DLL3）基因转录本，在SCLC患者血液样本中实现了60.8%-88.0%的阳性检测率，同时分析出这些指标与较短的OS有关，这提示CTCs在SCLC诊断方面具有较大的临床应用价值。

#### 2.4.2 外泌体

外泌体是直径为30-150 nm的细胞外囊泡，由多囊泡体与质膜融合分泌^[41]^。外泌体含有各种物质，如蛋白质、脂质和核酸，包括mRNA、非编码RNA和DNA^[42]^，该研究还表明，外泌体促进了肿瘤的进展，包括抗细胞凋亡、转移、血管生成、免疫逃避和化疗耐药性。Jimenez等^[43]^分析了SCLC细胞分泌的外泌体可促进SCLC细胞的黏附和存活。Mao等^[44]^研究了外泌体miRNA-375-3p在调节血管内皮屏障完整性和SCLC转移中的关键作用。SCLC来源的富含miR-375-3p的外泌体可以通过靶向血管claudin-1蛋白来破坏血液屏障，使SCLC转移更容易。因此，外泌体具有监测转移和指导SCLC患者临床治疗的潜力。

虽然上述生物标志物或可成为潜在的SCLC骨转移筛查、诊断的参考指标，但多数仍停留在实验室研究阶段，并未被广泛应用于临床中，且目前国内外尚未明确统一的检测标准，未来仍需要进行进一步探索。

## 3 小结

SCLC被业界称为“顽固的肿瘤”，属神经内分泌恶性肿瘤，可自分泌和外分泌生物因子促进肿瘤生长。不同于NSCLC中伴有表皮生长因子受体（epidermal growth factor receptor, EGFR）突变的腺癌，SCLC存在广泛的基因突变，是一种基因突变负荷较高的恶性肿瘤。众所周知，SCLC标准的治疗方案已有30-40年基本未做更改，“聪明”的肿瘤细胞在发生的时候总能巧妙地躲避免疫监视功能从而坐地生根。而SCLC骨转移的机制是一个复杂的过程，涉及多个信号和通路的参与，现有的研究主要集中在体外实验和小鼠模型，临床试验验证相对较少。笔者认为，SCLC未来研究方向应着重聚焦其生物学行为，探索更加有效的诊断方法和治疗方案，以实现精准医疗并最终提高患者的生存率和生活质量。针对SCLC的早期发现和风险评估，开发新的生物标志物和成像技术十分必要，这些技术包括但不局限于血液生物标志物、CTCs的定量分析以及基于人工智能的影像诊断系统等，力争早发现、早诊断、早治疗。通过对比分析，我们发现相关标志物具有取材简单，重复性好等优势，与此同时，在不同种族和不同癌种之间以及不同检测平台均存在临界值不统一的问题，且单个标志物的特异性、敏感性均有待提高，因此，联合检测和分析的结果尚需在临床进行进一步验证。未来，本课题组仍将继续聚焦SCLC骨转移相关的标志物及检测方法，运用前瞻性大型随机试验来更好地定义相关标志物，以期实现精准医疗时代下肺癌骨转移的早期筛查、动态预测和监测SCLC骨转移全过程，进而精确指导临床用药方案、病程管理及后续随访，积极预防相关的并发症，提高患者生存质量。
